# (*R,S*)-ketamine and (2*R,6R*)-hydroxynorketamine differentially affect memory as a function of dosing frequency

**DOI:** 10.1038/s41398-021-01685-0

**Published:** 2021-11-12

**Authors:** Lace M. Riggs, Xiaoxian An, Edna F. R. Pereira, Todd D. Gould

**Affiliations:** 1 Program in Neuroscience and Training Program in Integrative Membrane Biology, University of Maryland School of Medicine, Baltimore, MD 21201 USA; 2 Department of Psychiatry, University of Maryland School of Medicine, Baltimore, MD 21201 USA; 3 Department of Epidemiology and Public Health, Division of Translational Toxicology, University of Maryland School of Medicine, Baltimore, MD 21201 USA; 4 Department of Pharmacology, University of Maryland School of Medicine, Baltimore, MD 21201 USA; 5 Department of Anatomy and Neurobiology, University of Maryland School of Medicine, Baltimore, MD 21201 USA; 6 Veterans Affairs Maryland Health Care System, Baltimore, MD 21201 USA

**Keywords:** Depression, Hippocampus

## Abstract

A single subanesthetic infusion of ketamine can rapidly alleviate symptoms of treatment-resistant major depression. Since repeated administration is required to sustain symptom remission, it is important to characterize the potential untoward effects of prolonged ketamine exposure. While studies suggest that ketamine can alter cognitive function, it is unclear to what extent these effects are modulated by the frequency or chronicity of treatment. To test this, male and female adolescent (postnatal day [PD] 35) and adult (PD 60) BALB/c mice were treated for four consecutive weeks, either daily or thrice-weekly, with (*R,S*)-ketamine (30 mg/kg, intraperitoneal) or its biologically active metabolite, (*2R,6R*)-hydroxynorketamine (HNK; 30 mg/kg, intraperitoneal). Following drug cessation, memory performance was assessed in three operationally distinct tasks: (1) novel object recognition to assess explicit memory, (2) Y-maze to assess working memory, and (3) passive avoidance to assess implicit memory. While drug exposure did not influence working memory performance, thrice-weekly ketamine and daily (*2R,6R*)-HNK led to explicit memory impairment in novel object recognition independent of sex or age of exposure. Daily (*2R,6R*)-HNK impaired implicit memory in the passive-avoidance task whereas thrice-weekly (*2R,6R*)-HNK tended to improve it. These differential effects on explicit and implicit memory possibly reflect the unique mechanisms by which ketamine and (*2R,6R*)-HNK alter the functional integrity of neural circuits that subserve these distinct cognitive domains, a topic of clinical and mechanistic relevance to their antidepressant actions. Our findings also provide additional support for the importance of dosing frequency in establishing the cognitive effects of repeated ketamine exposure.

## Introduction

Depression is a common and often devastating neuropsychiatric disorder that is difficult to treat. Monoaminergic-based antidepressants take several months to exert a clinically significant therapeutic effect, though, many patients are prone to symptom relapse or fail to respond altogether [1]. In patients who are treatment-resistant, a subanesthetic dose of ketamine can rapidly alleviate symptoms of depression within hours of a single administration [1–3]. These antidepressant effects are transient, in that symptoms tend to return in the days or weeks immediately following the initial ketamine infusion. This has led to the implementation of a thrice-weekly ketamine administration paradigm to help sustain its antidepressant actions [4–7]. While the acute side effect profile of ketamine is well documented following a single [8–10] or repeated exposure [11], there is a limited controlled investigation into the enduring consequences of prolonged ketamine treatment [12].

Longitudinal observations suggest that frequent recreational use of ketamine (i.e., 4 or more days a week) is associated with a persistent form of cognitive impairment, which is absent in infrequent ketamine users (i.e., who use at least once a month, but not more than four times a week) and frequency-matched non-ketamine polydrug controls [13–15]. Acute cognitive deficits have also been reported in healthy volunteers following a single subanesthetic dose (0.5–0.65 mg/kg), which are transient and typically subside soon after drug cessation [16–18]. However, in patients with treatment-resistant major depression, the same dosing regimen has instead been shown to improve executive function, processing speed, and episodic memory when assessed a day after treatment [19]. Such pro-cognitive effects can last up to 7 days after a single administration [20] and appear to be sustained and enhanced by repeated exposure [21–23]. One reason why ketamine may exert these apparently dichotomous effects is that patients with depression often suffer from a form of cognitive impairment that recreational users and healthy volunteers do not [24–27]. The pro-cognitive effects of single and repeated ketamine (0.5 mg/kg, 40-min infusion) are at least in part related to its antidepressant actions [19] since improvements in cognitive function are correlated with a greater antidepressant response up to a week following infusion [23, 28–30]. These pro-cognitive effects appear to be unique to ketamine’s antidepressant mechanism of action [1] since the cognitive deficits that are associated with depression tend to persist even when patients respond favorably to traditional antidepressants [24, 25, 31]. Additional studies are needed to better understand the clinical factors that modulate the cognitive effects of ketamine, said to include age, sex, dose, route of administration, and length and frequency of drug treatment [22, 32].

As an *N*-methyl-d-aspartate receptor (NMDAR) antagonist [8], ketamine has been used as a pharmacological tool to model schizoaffective conditions that present with cognitive impairment via NMDAR hypofunction [18, 33, 34]. While NMDAR inhibition may explain why recreational use of ketamine is associated with cognitive deficits, it is unclear whether NMDAR inhibition accounts for the pro-cognitive effects of ketamine in patients with depression. A prevailing view in the field is that ketamine restores the functional integrity of neural circuits that are compromised in depression through a synaptogenic process that is triggered by the rapid activity-dependent release of brain-derived neurotrophic factor (BDNF) [1]. We reasoned that this may also confer ketamine with an ability to improve cognitive deficits that stem in part from circuit dysfunction. If the pro-cognitive effects of ketamine are due to its actions as an NMDAR antagonist, then these properties are not likely to be shared by its (*2R,6R*)-hydroxynorketamine (HNK) metabolite that has substantially less NMDAR-binding affinity [35, 36]. However, if both ketamine and (*2R,6R*)-HNK improve cognition, then a convergent mechanism unrelated to NMDAR inhibition is more likely to have given rise to these effects. For instance, (*2R,6R*)-HNK exerts preclinical antidepressant-like effects [36] by facilitating α-amino-3-hydroxy-5-methyl-4-isoxazolepropionic acid receptor (AMPAR)-mediated synaptic transmission independent of NMDAR activity or glutamatergic network disinhibition [35, 37]. We hypothesized that repeated exposure to ketamine and (*2R,6R*)-HNK will improve cognitive function through a persistent potentiation of the hippocampal activity or other metaplastic processes that enhances the efficacy of synaptic transmission. As an initial test of this hypothesis, we assessed changes in explicit, implicit, and working memory performance—discrete cognitive domains that are of clinical and mechanistic relevance to these compounds’ proposed antidepressant mechanism of action [1, 38]. By implementing a comprehensive experimental design that controls for an effect of age, sex, dosing frequency, and heterogeneity in the experimental outcome, we find that ketamine and (*2R,6R*)-HNK differentially affect memory as a function of dosing frequency.

## Materials and methods

### Animals

Male and female BALB/cAnNCrl (BALB/c) mice (Charles River Laboratories) were acclimated to the vivarium (University of Maryland, Baltimore, MD) for 1 week prior to experiments. Mice were housed five per cage under standard conditions (12-h light–dark cycle, lights on at 7:00 AM) with food and water available ad libitum. All experimental procedures were approved by the University of Maryland Baltimore Animal Care and Use Committee and were conducted in full accordance with the National Institutes of Health Guide for the Care and Use of Laboratory Animals.

### Drugs

(*R,S*)-ketamine (ketamine, Sigma-Aldrich; St. Louis, MO) hydrochloride was dissolved in pharmaceutical-grade sterile saline (0.9% NaCl) and administered at a dose of 30 mg/kg (intraperitoneal, (i.p.)) at a volume of 10 ml/kg. (*2R,6R*)-hydroxynorketamine (HNK) was provided by the National Center for Advancing Translational Sciences (Bethesda, MD), dissolved in pharmaceutical-grade sterile saline (0.9% NaCl), and administered at a dose of 30 mg/kg (i.p.) at a volume of 10 ml/kg. Vehicle (VEH)-treated mice received 10 ml/kg of the same sterile saline that was used to prepare the drug solutions for each experiment. Absolute and relative stereochemistry for (*2R,6R*)-HNK was confirmed by small-molecule X-ray crystallography, as previously described [39]. Doses are within the antidepressant dose–response range of ketamine and (*2R,6R*)-HNK [36] and were selected based on previous studies demonstrating cognitive effects of repeated ketamine exposure [22, 32, 33].

### Experimental design

**Fig. 1:**
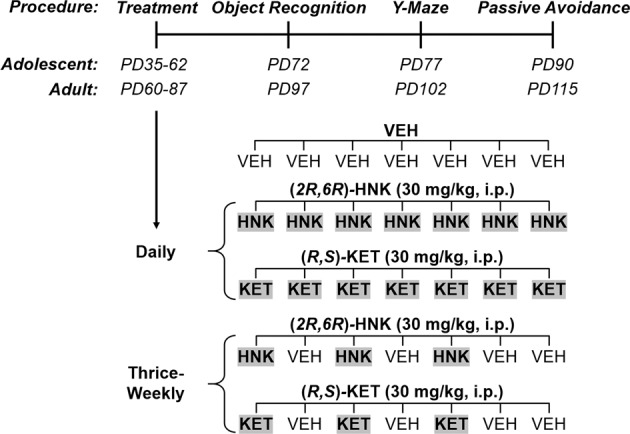
Experimental timeline. Male and female BALB/c mice were treated for 4 consecutive weeks during adolescence (postnatal day [PD] 35–62) or adulthood (PD 60–87) either thrice-weekly (M–W–F) or daily with either (*R,S*)-ketamine (KET) or its metabolite, (*2R,6R*)-hydroxynorketamine (HNK). For thrice-weekly drug administration, mice received a saline injection on Tuesday, Thursday, Saturday, and Sunday to control for the handling and stress of the injection. Memory performance was assessed beginning 10 days after drug cessation in three operationally distinct tasks: (1) novel object recognition to assess explicit memory, (2) Y-maze to assess working memory, and (3) passive avoidance to assess implicit memory.

Since thrice-weekly ketamine treatment appears to exert more pronounced pro-cognitive effects in patients with the anxious subtype of treatment-resistant major depression [40], we used the BALB/c mouse strain that is known to have a heightened anxiety-like phenotype [41]. In addition, our unpublished data suggest that BALB/c mice present with a more robust antidepressant-like response to ketamine and (*2R,6R*)-HNK (i.e., relative to other mouse strains) under certain experimental conditions. All mice received a single injection per day for 28 consecutive days, and behavioral testing began 10 days after the last day of injections (Fig. 1). This was to ensure that we would only measure sustained effects of chronic treatment, rather than the drugs’ acute effects or their immediate sequelae. To determine whether drug exposure modulates learning and memory performance as a function of age, treatment began either during adolescence (postnatal day [PD] 35–62) or adulthood (PD 60–87). Mice were treated either daily or thrice-weekly to assess whether dosing frequency modulates learning and memory outcomes. For the daily drug administration condition, mice were treated with either saline, ketamine, or (*2R,6R*)-HNK once per day. For thrice-weekly drug administration, mice were treated with either ketamine or (*2R,6R*)-HNK on Monday, Wednesday, and Friday, and received a saline injection on Tuesday, Thursday, Saturday, and Sunday to control for the handling and stress of the injection (Fig. 1). Testing was conducted during the light phase and in order of the least-to-most stressful procedure. Mice were acclimated to the testing room for one hour prior to the onset of any behavioral procedures and behavioral equipment was cleaned in between each animal using MB-10 solution and allowed to dry fully prior to the start of the next trial.

### Novel object recognition

The novel object-recognition (NOR) task was used to assess short-term recognition memory [42]. The NOR apparatus (40-cm width × 40-cm depth × 35-cm height; Stoelting, IL) consisted of two adjacent test chambers (20-cm width × 18-cm depth) that were joined by a start box (20-cm width × 10-cm depth). NOR was conducted under dim-yellow lighting conditions, with the center of each chamber receiving ~10–15 lux. During the 30-min habituation phase, mice were placed into the start box and allowed to freely explore the NOR apparatus in the absence of any objects. Habituation was immediately followed by a 30-min training phase, in which mice freely explored two identical objects that were placed in the center of each test chamber. The objects consisted of either two 50-mL clear glass flasks (4.5-cm width × 7-cm height) or two white glass vials (2.5-cm width × 6-cm height) and were counterbalanced between groups. After training, mice were returned to their home cage for 60 min until testing took place. During testing, mice were placed back into the NOR apparatus, which now contained a novel object (i.e., that they had never encountered) and a familiar object (i.e., that they had encountered during training), and were allowed to explore for 10 min. The side in which the novel object was placed was counterbalanced between groups and the novel object (i.e., either the clear glass flask or the white glass vial) was randomized between groups (i.e., according to which objects they observed during training). All phases of the procedure were recorded by an overhead digital camera and analyzed using CleverSys Inc Software (Reston, VA). Distance traveled during habituation was determined with the CleverSys Inc TopScan center-point detection module and was used to assess general locomotor activity. Time spent interacting with each object during training was also determined with TopScan center-point detection and was used to establish baseline criteria for training exploration and to rule out potential side-preferences. Time spent interacting with each object during testing was determined with the CleverSys Inc AnnoStar Behavioral Annotation module, in which an experimenter blinded to the treatment groups entered keystrokes to record active exploration, defined as nose-oriented interactions with either of the two objects. The discrimination index was calculated as the time spent with the novel object minus the time spent with the familiar object divided by total object exploration time (i.e., (novel-familiar)/(novel+familiar)). A higher discrimination index reflects a greater amount of time exploring the novel object relative to the familiar object. If mice fail to recognize that they previously encountered the familiar object, they will have a reduced discrimination index.

### Y-maze

The Y-maze task was used to assess spontaneous working memory [42] and was conducted using a Y-shaped apparatus that has three identical arms (5-cm width × 35-cm length) spaced 120° (Stoelting, IL). The task was conducted under dim-yellow lighting conditions, with the interior of each arm receiving ~35–40 lux. Mice were placed into one of the three arms of the maze in a randomized fashion and were allowed to explore the maze for 8 min. Arm entries were scored with the AnnoStar Behavioral Annotation module. Specifically, an experimenter blinded to treatment groups entered keystrokes to record an individual arm entry (i.e., when all four limbs are within a given arm). The total number and sequence of arm entries were used to assess percent alternations (i.e., (the number of alternations/total arm entries − 2) × 100), defined as consecutive entry into three different arms (i.e., ABC as opposed to ABA). Mice tend to enter arms that are not recently visited, thus, a reduction in spontaneous alternations reflects a diminished spatial novelty preference in spontaneous exploration [42].

### Passive avoidance

The passive-avoidance task was used to assess threat-aggravated memory [42]. A light–dark shuttle box was used (Coulbourn Instruments, PA), which contains two chambers (16-cm width × 18-cm depth × 34-cm height) that are separated by a wall and automated guillotine door. During training, mice were placed into the light compartment (~800 lux) while the center guillotine door remained shut. After 30 s of being in the light compartment, the guillotine door opened, and mice were given 10 min to cross over to the dark compartment. Once mice crossed into the dark compartment, the guillotine door shut, and an inescapable foot shock (0.5 mA, 2-s duration) was delivered 3 s later. Mice were returned to their home cage 30 s after termination of the foot shock. Testing took place 24 h later, at which time mice were reintroduced into the light compartment. After 30 s of being in the light compartment, the guillotine door was opened, and mice were given 10 min to cross over to the dark compartment. If and when mice crossed over into the dark compartment, the guillotine door shut but no foot shock was delivered, and 30 s later, mice were returned to their home cage. The latency to enter the dark compartment during training and testing was detected automatically by a photocell response sensor within the shuttle box and recorded by the Graphic State Notation Software (Coulbourn Instruments, PA). Because mice prefer darkened enclosures, they will readily cross to the dark compartment during training. If mice remember that entry into the dark compartment was associated with the aversive foot shock, they will have a longer latency to cross into the dark compartment during testing. A shorter latency to cross during testing reflects an impairment in threat-aggravated implicit memory [42].

### Statistical analyses

Data were analyzed with GraphPad Prism Software 9.0.1 and assessed for normality (D’Agostino–Pearson) and homogeneity of variance (corrected Bartlett’s test). Data are presented as mean ± standard error of the mean and statistical significance was defined as *P* < 0.05. When parametric assumptions were met, between-group comparisons of three or more groups were assessed using one-way analysis of variance (ANOVA) followed by Holm–Šídák post hoc comparisons. When parametric assumptions were not met, between-group comparisons of three or more groups were assessed using the Kruskal–Wallis test followed by Dunn’s multiple comparisons. Two-way repeated-measures ANOVA was used when drug condition (VEH vs. thrice-weekly ketamine vs. thrice-weekly (*2R,6R*)-HNK vs. daily ketamine vs. daily (*2R,6R*)-HNK) and time (5-min time bins, repeated measure) or object (novel vs. familiar; repeated measure) were independent factors. If a significant main effect or interaction was detected, the Holm–Šídák post hoc test was used to assess pairwise comparisons. Frequency differences were assessed using the chi-squared test followed by Bonferroni correction for multiple comparisons. Score distribution/survival plots were assessed using the Kaplan–Meier survival analysis followed by Mantel–Cox log-rank chi-squared test with Bonferroni correction.

### Rigor and reproducibility

All experiments were performed in a randomized fashion and were conducted and analyzed by experimenters who were blind to the treatment conditions. To minimize and reduce the number of animals needed to assess drug effects as a function of sex, age, drug, and dosing frequency, we conducted behavioral testing in sequence within each experiment as opposed to using independent sets of animals to test each behavior. Experiments were performed in order from least to more stressful so that it was unlikely that prior testing would influence the results of subsequent tests. To account for potential housing effects, drug conditions were randomly assigned across cages, with each of the five conditions equally represented within each cage (i.e., each mouse, of five total within a cage, was assigned to one of the five drug conditions). An equal number of male and female mice were assigned to each of the drug conditions (*n* determined by power analyses, pilot experiments, and published literature) within a given age group and were run as sequential experiments: (1) adolescent females (*n* = 8/drug, *N* = 40), (2) adolescent males (*n* = 8/drug, *N* = 40), (3) adult females (*n* = 8/drug, *N* = 40), and (4) adult males (*n* = 8/drug, *N* = 40). Due to a technical issue, the Y-maze and passive avoidance results from the adult-treated females could not be reliably compared to the other experimental groups and were thus not included in the final analyses.

Individual data points that correspond to male and female data are colored in pink and blue, respectively, throughout the manuscript figures. Mice were only excluded from the final analyses if they (a) did not interact with the identical objects during training in the NOR task or (b) retreated back into the bright chamber after triggering the guillotine door during training in the passive-avoidance task. In the adult-treated conditions, five mice did not markedly interact with the objects during the training phase of the novel object-recognition task, as their cumulative exploration time was less than 100 s over the 30 min allotted (VEH, *n* = 3; thrice-weekly ketamine, *n* = 1; daily ketamine, *n* = 1), and thus their data were excluded from the final analyses. In the training phase of the passive-avoidance task, nine of the adolescent-treated mice retreated back into the bright chamber after triggering the guillotine door (VEH, *n* = 3; thrice-weekly ketamine, *n* = 2; thrice-weekly (*2R,6R*)-HNK, *n* = 2; daily (*2R,6R*)-HNK, *n* = 2) and three of the adult-treated mice retreated back into the bright chamber after triggering the guillotine door (thrice-weekly (*2R,6R*)-HNK, *n* = 1; daily ketamine, *n* = 1; daily (*2R,6R*)-HNK, *n* = 1). As these mice did not receive the foot shock in the dark chamber, their data were excluded from the final analyses. In the few instances in which statistically significant outliers were detected by ROUT (*Q* = 1%), data were analyzed with and without outliers using parametric and nonparametric alternatives, and both are described in “Results”.

## Results

### Body weight is not influenced by (*R,S*)-ketamine or (*2R,6R*)-hydroxynorketamine regardless of sex or age of exposure

**Fig. 2:**
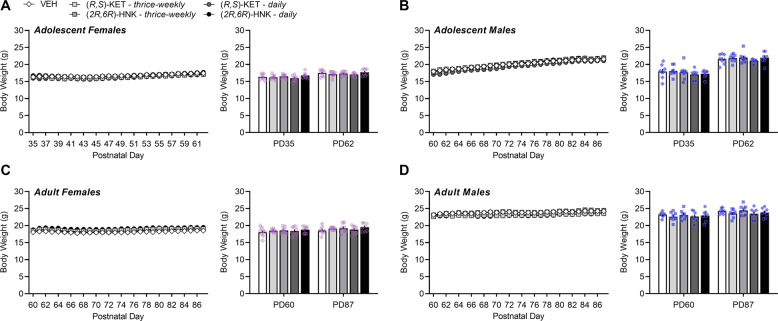
Body weight is not influenced by (*R,S*)-ketamine or (*2R,6R*)-hydroxynorketamine regardless of sex or age of exposure. Body weight of adolescent female (**A**), adolescent male (**B**), adult female (**C**), and adult male (**D**) BALB/c mice over 4 weeks of treatment: 0.9% saline (vehicle, VEH; white/diamonds), thrice-weekly (*R,S*)-KET (light-gray/square), thrice-weekly (*2R,6R*)-HNK (dark-gray/square), daily (*R,S*)-KET (dark-gray/circle), daily (*2R,6R*)-HNK (black/circle). Data are presented as mean ± standard error of the mean.

There was a significant main effect of sex and age on body weight when included as an independent factor in the analyses, and thus body weight data were separated by age and sex. Prior to treatment, body weight varied significantly as a function of sex (*F*_(1,140)_ = 204.00, *P* < 0.0001) and age (*F*_(1,140)_ = 343.80, *P* < 0.0001) independent of later drug condition assignment (*F*_(4,140)_ = 0.65, *P* = 0.6256), which yielded a significant sex × age interaction (*F*_(1,140)_ = 64.77, *P* < 0.0001). Adults of both sexes weighed more than adolescents (*F*_(1,150)_ = 124.80, *P* < 0.0001), and males of both ages weighed more than females (*F*_(1,150)_ = 55.51, *P* < 0.0001). After 28 days of drug exposure, differences in body weight still varied significantly as a function of sex (*F*_(1,140)_ = 656.10, *P* < 0.0001) and age (*F*_(1,140)_ = 116.60, *P* < 0.0001) and was not further influenced by drug condition (*F*_(4,140)_ = 1.76, *P* = 0.1409). When assessing changes in body weight over the course of treatment within age and sex (Fig. 2), there was a significant main effect of postnatal day in adolescent-treated mice (*P* < 0.0001) independent of drug condition in both females (*F*_(4,35)_ = 1.625, *P* = 0.1897; Fig. 2A) and males (*F*_(4,35)_ = 0.7292, *P* = 0.8781; Fig. 2B). Likewise, there was a significant main effect of postnatal day on body weight in adult-treated mice (*P* < 0.0001) independent of drug condition in both females (*F*_(4,35)_ = 1.144, *P* = 0.3521; Fig. 2C) and males (*F*_(4,35)_ = 0.9039, *P* = 0.4723; Fig. 2D). Thus, treatment with either ketamine or (*2R,6R*)-HNK did not influence body weight in adolescent- or adult-treated male and female BALB/c mice.

### Adolescents treated with thrice-weekly (*R,S*)-ketamine or daily (*2R,6R*)-hydroxynorketamine have impaired novel object recognition in adulthood

**Fig. 3:**
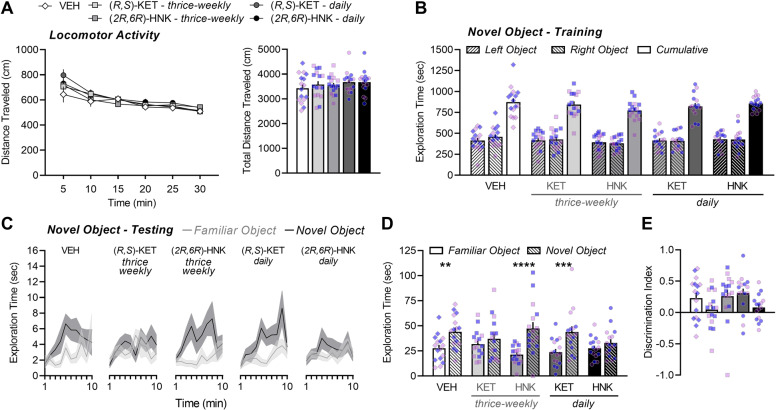
Adolescents treated with thrice-weekly (*R,S*)-ketamine or daily (*2R,6R*)-hydroxynorketamine have impaired novel object recognition in adulthood. **A** Locomotor activity during the habituation phase of the novel object-recognition task (i.e., in the absence of objects) plotted as 5-min time bins (left) and summarized as total distance traveled over the total 30-min (right). **B** Total time spent exploring two identical objects during the 30-min of training. **C** Time spent exploring the familiar object and the novel object across the 10-min of testing. **D** Total time spent exploring the familiar object and the novel object during the 10-min of testing and (**E**) plotted as a discrimination index (right): (novel-familiar)/(novel + familiar). Data are presented as mean ± standard error of the mean. **P < 0.01, ***P < 0.001, ****P < 0.0001.

There was a significant main effect of age on object exploration during training (*F*_(1,131)_ = 8.05, *P* = 0.0053) and testing (*F*_(1,131)_ = 7.99, *P* = 0.0054), and thus data from adolescent- and adult-treated mice were analyzed separately. We did not observe a main effect of sex during training (*F*_(1,131)_ = 1.60, *P* = 0.2080) or testing (*F*_(1,131)_ = 0.2205, *P* = 0.6394), and thus data from males and females were combined. In adolescent-treated male and female mice, distance traveled during the 30-min habituation phase was used to assess general locomotor activity. All mice habituated to the open-field environment, as evidenced by a significant main effect of time (*P* < 0.0001; Fig. 3A, left). Treatments had no significant effect on distance traveled throughout the habituation phase (*P* = 0.5909; Fig. 3A, left) or on cumulative distance traveled overall (*F*_(4,75)_ = 0.7048, *P* = 0.5911; Fig. 3A, right). During training, mice spent an equivalent amount of time exploring the identical objects that were placed in the left and right compartment (object main effect: *F*_(1,75)_ = 0.1431, *P* = 0.7063), which was not influenced by drug exposure (drug main effect: *F*_(4,75)_ = 1.212, *P* = 0.3127; Fig. 3B). When assessing exploration time across the testing procedure (Fig. 3C), a significant main effect of object was observed in mice treated with VEH (*F*_(1,15)_ = 8.905, *P* = 0.0093), daily ketamine (*F*_(1,15)_ = 15.52, *P* = 0.0013), and thrice-weekly (*2R,6R*)-HNK (*F*_(1,15)_ = 19.07, *P* = 0.0006). Drug exposure led to a rightward shift in the peak time of novel object exploration, with daily ketamine and thrice-weekly (*2R,6R*)-HNK*-*treated mice spending the greatest amount of time inspecting the novel object at 9 min and 8 min into the procedure, respectively, compared to at 6 min for VEH-treated mice (Fig. 3C). In contrast, mice from the thrice-weekly ketamine (*F*_(1,15)_ = 0.8792, *P* = 0.3633) and daily (*2R,6R*)-HNK (*F*_(1,15)_ = 2.997, *P* = 0.1040) conditions spent a similar amount of time exploring each object during the testing phase (Fig. 3C). Consistent with this, there was a significant main effect of object (*F*_(1,75)_ = 40.46, *P* < 0.0001) and a significant object × drug interaction (*F*_(4,75)_ = 3.151, *P* = 0.0189) when assessing total exploration time overall (Fig. 3D), which was driven by significant novel object exploration in mice treated with VEH (*P* = 0.0061), thrice-weekly (*2R,6R*)-HNK (*P* < 0.0001), or daily ketamine (*P* = 0.0008). In contrast, mice in the thrice-weekly ketamine and daily (*2R,6R*)-HNK conditions spent the same amount of time exploring the novel and familiar object (*P* = 0.4967, respectively), indicating that these drug conditions led to a sustained impairment in recognition memory. Deficits in novel object recognition were further evidenced as a near-significant reduction in discrimination index scores with regard to the main effect of treatment (Fig. 3E; *F*_(4,75)_ = 2.169, *P* = 0.0806). The impairment in novel object recognition could not be explained by differences in the amount of familiarization during training, as cumulative object exploration was not significantly different among the groups (Fig. 3B) nor correlated with novel object exploration during testing (data not shown). Thus, prolonged exposure to thrice-weekly ketamine or daily (*2R,6R*)-HNK impairs explicit recognition memory in adolescent-treated male and female BALB/c mice.

### Adults treated with thrice-weekly (*R,S*)-ketamine or daily (*2R,6R*)-hydroxynorketamine have impaired novel object recognition later in adulthood

**Fig. 4:**
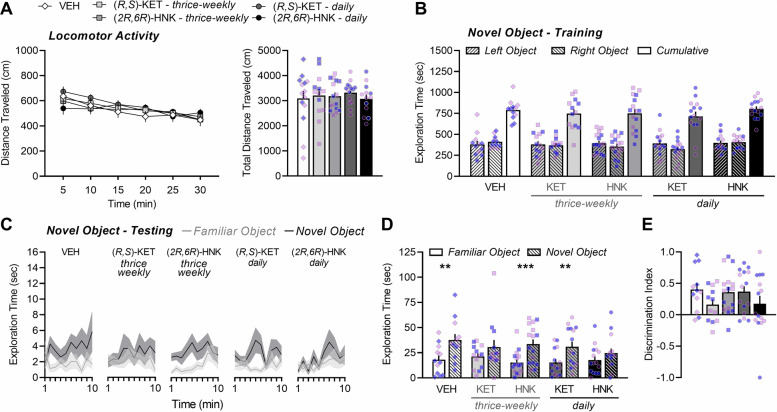
Adults treated with thrice-weekly (*R,S*)-ketamine or daily (*2R,6R*)-hydroxynorketamine have impaired novel object recognition later in adulthood. **A** Locomotor activity during the habituation phase of the novel object-recognition task (i.e., in the absence of objects) plotted as 5-min time bins (left) and summarized as total distance traveled over the total 30 min (right). **B** Total time spent exploring two identical objects during the 30 min of training. **C** Time spent exploring the familiar object and the novel object across the 10 min of testing. **D** Total time spent exploring the familiar object and the novel object during the 10 min of testing and (**E**) plotted as a discrimination index (right): (novel-familiar)/(novel + familiar). Data are presented as mean ± standard error of the mean. **P < 0.01, ***P < 0.001.

Adult-treated male and female mice habituated to the open-field environment, as evidenced by a significant main effect of time (*P* < 0.0001; Fig. 4A, left). Treatment had no significant effect on distance traveled throughout the habituation phase (*P* = 0.9333; Fig. 4A, left) or on the cumulative distance traveled overall (*F*_(4,66)_ = 0.2604, *P* = 0.9023; Fig. 4A, right). During training, mice spent an equivalent amount of time exploring the identical objects that were placed in the left and right compartment (object main effect: *F*_(1,66)_ = 0.7634, *P* = 0.3854), and exploration was not influenced by drug exposure (drug main effect: *F*_(4,66)_ = 0.5921, *P* = 0.6696; Fig. 4B). When assessing exploration time across the testing procedure (Fig. 4C), a significant main effect of object was observed in mice treated with VEH (*F*_(1,11)_ = 12.70, *P* = 0.0044), thrice-weekly (*2R,6R*)-HNK (*F*_(1,15)_ = 19.30, *P* = 0.0005), and daily ketamine (*F*_(1,14)_ = 13.18, *P* = 0.0027). Drug exposure led to a leftward shift in the peak time of novel object exploration, with thrice-weekly (*2R,6R*)-HNK and daily ketamine-treated mice spending the greatest amount of time with the novel object at 8 min into the procedure compared to at 10 min for VEH-treated mice. In contrast, mice from the thrice-weekly ketamine (*F*_(1,12)_ = 2.654, *P* = 0.1292) and daily (*2R,6R*)-HNK (*F*_(1,14)_ = 2.914, *P* = 0.1099) conditions spent a similar amount of time exploring each object (Fig. 4C). Consistent with this, there was a significant main effect of object (*F*_(1,66)_ = 43.44, *P* < 0.0001) when assessing total exploration time overall (Fig. 4D), which was driven by significant novel object exploration in mice treated with VEH (*P* = 0.0013), thrice-weekly (*2R,6R*)-HNK (*P* < 0.0006), and daily ketamine (*P* = 0.0031). In contrast, mice in the thrice-weekly ketamine and daily (*2R,6R*)-HNK conditions spent the same amount of time exploring the novel and familiar object (*P* = 0.1152 and 0.1295, respectively), a finding that suggests these drug conditions led to a sustained impairment in recognition memory. The main effect of treatment on discrimination index did not reach statistical significance (Fig. 4E; *F*_(4,65)_ = 1.344, *P* = 0.2630) though similar to adolescent-treated mice, the mean discrimination index was numerically lower for mice treated thrice-weekly with ketamine or with daily (*2R,6R*)-HNK compared to control mice. The impairment in NOR could not be explained by differences in the amount of familiarization during training, as cumulative object exploration was not significantly different among the groups (Fig. 4B) nor correlated with novel object exploration during testing (data not shown). Thus, prolonged exposure to thrice-weekly ketamine or daily (*2R,6R*)-HNK impairs explicit recognition memory in adult-treated male and female BALB/c mice.

### Working memory is not influenced by (*R,S*)-ketamine or (*2R,6R*)-hydroxynorketamine regardless of sex or age of exposure

**Fig. 5:**
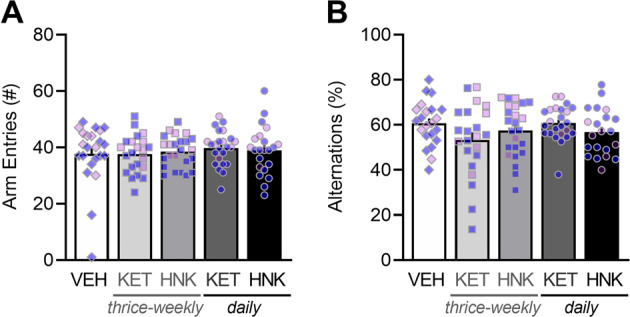
Working memory is not influenced by (*R,S*)-ketamine or (*2R,6R*)-hydroxynorketamine regardless of sex or age of exposure. **A** Total arm entries and **B** percent alternations ((the number of alternations/total arm entries − 2) × 100) during the 8-min task. Data are presented as mean ± standard error of the mean.

In the Y-maze task, we did not observe a main effect age (*F*_(1,66)_ = 2.19, *P* = 0.1431) or sex (*F*_(1,69)_ = 2.14, *P* = 0.1485), and thus data from adolescent- and adult-treated male and female mice were combined. Standard deviations for the number of arm entries were significantly different (Bartlett’s = 13.39, *P* = 0.0095), with the difference being driven by the presence of two significant outliers in the VEH-treated group. Upon removal, standard deviations among the groups were normalized (Bartlett’s = 6.183, *P* = 0.5673) and parametric comparisons revealed that the number of arm entries did not vary among the groups (*F*_(4,108)_ = 0.6612, *P* = 0.6203; Fig. 5A). Similar results were found with nonparametric tests when outliers were included (*P* = 0.6203), and thus the number of arm entries was not influenced by drug condition regardless of outlier handling. Similar, standard deviations for percent alternations were significantly different (Bartlett’s = 13.93, *P* = 0.0075) and driven in part by the presence of one significant outlier each in the VEH- and thrice-weekly (*2R,6R*)-HNK*-*treated groups. Upon removal, standard deviations among the groups were not normalized (Bartlett’s = 13.16, *P* = 0.0105) and so nonparametric comparisons were made, which revealed that percent alternations did not vary among the groups (*P* = 0.1711; Fig. 5B). Thus, prolonged exposure to ketamine or (*2R,6R*)-HNK does not influence spatial working memory in adolescent- or adult-treated male and female BALB/c mice.

### Daily (*2R,6R*)-hydroxynorketamine treatment impairs implicit memory in the passive-avoidance task while thrice-weekly treatment tends to improve it

**Fig. 6:**
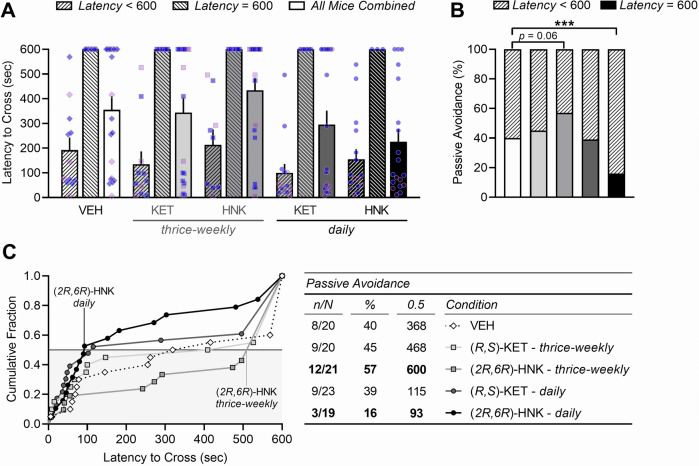
Daily (*2R,6R*)-hydroxynorketamine treatment impairs implicit memory in the passive-avoidance task while thrice-weekly treatment tends to improve it. **A** Latency to cross into the dark shock-paired chamber during testing. Mice that did not cross during the 10-min procedure (latency = 600 sec) display (**B)** “complete” passive avoidance, defined as a failure to cross within 600 s. **C** Drug exposure led to a shift in the cumulative fraction of scores with regard to latency to cross; corresponding values represent the number of animals that did not cross within each condition (*n*/N), the percentage of animals that did not cross within each condition (%), and the 50th percentile of scores within each condition (0.5). Data are presented as mean ± standard error of the mean. ***P < 0.001.

In the passive-avoidance task, we did not observe a main effect age (*F*_(1,66)_ = 1.12, *P* = 0.2933) or sex (*F*_(1,65)_ = 0.06, *P* = 0.8021), and thus data from adolescent- and adult-treated male and female mice were combined. Mice of all treatment groups had a similar latency to cross into the darkened compartment during the training phase of the passive-avoidance task, which occurred on average within 40 s of trial onset (*F*_(4,98)_ = 0.7099, *P* = 0.5871). This suggests that drug exposure did not alter baseline preference for the darkened enclosure prior to foot shock. However, entry-based delivery of a single foot shock led to passive avoidance of the darkened compartment on a subsequent day (*F*_(1,98)_ = 157.20, *P* < 0.0001; Fig. 6A). Interestingly, there was significant heterogeneity in animals’ propensity to cross during testing (D’Agostino–Pearson = 576.50, *P* < 0.0001), with 60% of mice crossing within 160 s of trial onset whereas 40% did not (*F*_(1,93)_ = 247.60, *P* < 0.0001). The proportion of mice that displayed “complete” passive avoidance, defined as a failure to cross within 10 min, varied significantly by drug condition (*χ*² = 37.24, *P* < 0.0001; Fig. 6A). Thrice-weekly ketamine (45%, *n* = 9/20) and daily ketamine (39%, *n* = 9/23) led to a comparable proportion of mice to display complete passive avoidance relative to VEH (40%, *n* = 8/20). In contrast, exposure to thrice-weekly (*2R,6R*)-HNK led to a greater proportion of mice to display complete avoidance (57%, *n* = 12/21, *P*_uncorrected_ = 0.016, *P* = 0.0648), whereas daily exposure to (*2R,6R*)-HNK led to a lesser proportion (16%, *n* = 3/19, *P* = 0.0008; Fig. 6B). This is further evidenced by a shift in the cumulative fraction of scores, in which over 50% of mice treated thrice-weekly with (*2R,6R*)-HNK did not cross whereas 50% of daily (*2R,6R*)-HNK-treated mice crossed within 93 s, yielding a significant separation between these two groups (*χ*² = 7.744, *P* = 0.027; Fig. 6C). While daily ketamine exposure tended to reduce the mean latency to cross into the dark compartment (115 s), the distribution of scores was comparable between thrice-weekly ketamine (*χ*² = 0.0037, *P* > 0.05) and daily ketamine (*χ*² = 0.2921, *P* > 0.05) relative to VEH (Fig. 6C). Thus, prolonged thrice-weekly (*2R,6R*)-HNK exposure promotes implicit memory in male and female BALB/c mice whereas daily (*2R,6R*)-HNK tends to impair it.

While estrous cycle was not monitored across the days of treatment or during testing, Bartlett’s test indicated that variance did not differ as a function of sex across the behaviors tested, which suggests that a factor specific to one sex (such as estrous cycle in females) did not markedly influence experimental outcomes in this study [43].

## Discussion

A single infusion of a subanesthetic dose of ketamine rapidly alleviates symptoms of depression in patients who do not respond to traditional antidepressants [1]. Considering that repeated administration is often required to sustain this remission [44], it is important to characterize the potential untoward effects of prolonged ketamine exposure. While a number of studies suggest that ketamine may dynamically regulate cognitive function following single or repeated administration [13, 21, 22, 32, 45], less is known regarding the persistence of these effects and to what extent they are modulated by the frequency or chronicity of treatment. To address this, we compared the sustained effects of prolonged exposure to ketamine and its (*2R,6R*)-HNK metabolite when administered either thrice-weekly or daily, on explicit, implicit, and working memory performance in male and female adolescent- and adult-treated BALB/c mice. Our data reveal that thrice-weekly treatment with ketamine leads to a sustained impairment in explicit recognition memory independent of age or sex—an effect that is also observed following daily treatment with (*2R,6R*)-HNK. In contrast, while ketamine did not influence implicit memory in the passive-avoidance task, performance was bidirectionally modulated by (*2R,6R*)-HNK depending on the frequency of drug administration. Neither ketamine nor (*2R,6R*)-HNK influenced working memory, possibly reflecting a cognitive domain that is insensitive to the long-term effects of these compounds.

Our results agree with previous preclinical studies examining the effects of repeated ketamine on recognition memory [33, 46–49] and further corroborate some clinical observations following single or repeated ketamine in healthy volunteers and recreational users [13, 14, 50–52]. However, while preclinical studies report on chronic ketamine-related deficits in spatial memory [53–55] and attention [56–58], we did not observe a robust effect of drug treatment on spontaneous alternations in the Y-maze. One reason for this apparent discrepancy is that ketamine may impair working memory through a transient, dose-dependent inhibition of memory consolidation in this task [59] that may not extend beyond drug cessation. Likewise, we did not observe a sustained effect of ketamine on threat-aggravated implicit memory in the passive-avoidance task, though deficits have been previously reported in this procedure shortly after single [60] or repeated [61] ketamine exposure. Our results, therefore, suggest that repeated ketamine administration may lead to longer-term deficits in a discrete set of cognitive domains that involve the storage and retrieval of explicit memory (e.g., object recognition), whereas deficits in working memory (e.g., spontaneous alternations) and implicit memory (e.g., threat-related) are relatively short-lived. It is important to consider, however, that explicit memory of novelty recognition requires the collective participation of the hippocampus, perirhinal cortex, and other associated brain regions [62–64], and thus no one region is solely responsible for performance in this task. Considering that sustained deficits in posterior perceptual memory have not been reported following ketamine exposure, future studies may further establish to what extent hippocampal microcircuitry is involved in the novelty recognition deficits induced by prolonged ketamine exposure.

An unexpected observation in the current study, is that (*2R,6R*)-HNK tended to improve implicit memory when administered thrice-weekly, yet the opposite was true following daily exposure. Acquiring passive avoidance involves an increase in the efficacy of synaptic transmission at Schaffer collateral hippocampal synapses—a long-term potentiation-like process that requires *N-*methyl-d-aspartate receptor (NMDAR) activation [65]. We have previously reported that (*2R,6R*)-HNK promotes a rapid potentiation of α-amino-3-hydroxy-5-methyl-4-isoxazolepropionic acid receptor (AMPAR)-mediated activity in the hippocampus by increasing the probability of glutamate release at Schaffer collateral synapses [37]. This is hypothesized to initiate a synaptogenic process that involves a delayed upregulation of synaptic AMPARs several hours later, possibly sustaining its antidepressant effects [1, 38]. This process could occlude further potentiation of those synapses by learned experiences that also depend on AMPAR upregulation as an expression mechanism. Indeed, similar to (*2R,6R*)-HNK, passive avoidance is associated with an upregulation of hippocampal AMPARs, which occludes further potentiation by high-frequency electrical stimulation of Schaffer collateral afferents [65]. Our results are consistent with an occlusive effect (*2R,6R*)-HNK on passive avoidance, particularly given that dosing frequency had a bidirectional effect on performance in this task. However, there are alternative explanations worth considering.

For instance, preclinical studies report that (*2R,6R*)-HNK promotes offensive aggression through its ability to potentiate glutamatergic synaptic transmission in the ventrolateral periaqueductal gray [66–68]. While these effects were observed after a single drug exposure, it is interesting to consider how an increased tendency to approach threat might influence performance in the passive-avoidance task. Alternatively, it is possible that different conclusions may be drawn if certain aspects of the experimental design were changed. For instance, we note that data were biphasically distributed, with mice either crossing into the shock-paired chamber early during testing or not at all. We addressed this heterogeneity using statistical approaches, which revealed that the proportion of mice who remembered the shock-paired chamber varied as a function of drug condition. However, some variations to the procedure can potentially overcome the issues of heterogeneity in our sample. To assess a deficit in passive avoidance, a stronger shock intensity could be used (i.e., to elicit passive avoidance in 100% of control mice). Initially, we avoided using a stronger stimulus, since ceiling effects could potentially mask a reduction in passive avoidance induced by drug exposure. However, given that thrice-weekly (*2R,6R*)-HNK promotes passive avoidance, a subthreshold shock (i.e., that control mice would not typically learn to avoid) could potentially reveal the true effect size of this observation. Nonetheless, the apparent selectivity of (*2R,6R*)-HNK relative to ketamine on the extent of passive avoidance may reflect distinct circuit-level mechanisms that are uniquely evoked by each compound. Currently, very little is known about how the cellular or circuit actions of (*2R,6R*)-HNK distinguish it mechanistically from ketamine [8, 38].

The critical role of NMDAR activation in passive avoidance may explain why ketamine impairs implicit memory shortly after drug exposure. However, unlike (*2R,6R*)-HNK, we did not observe a sustained effect of ketamine on the propensity of mice to avoid the shock-paired chamber during testing. Using a twenty-eight-day regimen as described in this study, Luo et al. [69] showed that, immediately following the continuous treatment of C57BL/6 mice with ketamine (30 mg/kg/day, i.p.), expression of synaptic and extrasynaptic AMPARs and NMDARs in the hippocampus is significantly reduced. They also found that dendritic complexity, glutamatergic transmission, and theta-burst-induced synaptic plasticity were impaired at Schaffer collateral synapses [69]. Similarly, the impairment in novel object recognition and location memory detected following repeated ketamine administration (40 mg/kg/day, i.p.) to ICR mice was associated with impaired theta-burst stimulation-evoked long-term potentiation at Schaffer collateral synapses, an effect that was blocked by the NMDAR glycine site partial agonist, N,N-dimethylglycine [70]. Thus, it is possible that reductions in glutamate receptor expression by ketamine are sufficient to counterbalance the potentially sustained potentiation of synaptic transmission induced by (*2R,6R*)-HNK following its in vivo metabolism from daily ketamine [37, 38]. While future work is needed to definitively test the role of NMDAR blockade in this process, continuous NMDAR inhibition likely mediates the effects of ketamine on receptor expression and synaptic plasticity [69]. This may also explain why the occlusion of passive avoidance was specific to (*2R,6R*)-HNK in this study, as this metabolite does not inhibit NMDAR at brain concentrations relevant to the dose tested here [35, 38].

The repeated administration of ketamine has served as an attractive model for conditions that are characterized by dissociative symptoms and cognitive impairment that are due to NMDAR hypofunction (e.g., schizophrenia [18, 50, 71]), often assayed preclinically as a deficit in NOR performance [72]. Interestingly, the acute hyperlocomotor response to ketamine that is triggered by NMDAR inhibition is more pronounced in preweanlings [73] and adolescents [74, 75] relative to adults, and in females relative to males, especially during adolescence [76, 77]. One interpretation of these observations is that younger rodents and females have an enhanced sensitivity to the NMDAR-inhibition-dependent actions of ketamine. If the recognition memory impairment we observed were due principally to NMDAR inhibition by ketamine, then our results would have mirrored this pattern of enhanced sensitivity in younger rodents and females. The fact that they did not suggest that these locomotor differences arise from distinctions in monoaminergic signaling downstream of NMDAR inhibition [73, 76], or alternatively, that the recognition impairments are not due to NMDAR inhibition. Consistent with the latter, we also observed recognition deficits following repeated (*2R,6R*)-HNK administration, a finding that suggests that sustained elevations in glutamate release, independent of NMDAR blockade or glutamatergic network disinhibition [37], is sufficient to alter explicit memory in NOR. This could potentially reconcile why daily ketamine apparently rescues the deficits induced by thrice-weekly ketamine since daily ketamine administration could block the deleterious effects of excess glutamatergic signaling via NMDARs during a time when the glutamate burst has been shown to manifest. Indeed, repeated administration of ketamine increases astrocyte proliferation and decreases glial-specific excitatory amino acid transporter expression, leading to sustained cognitive dysfunction via impaired reuptake of excess hippocampal glutamate [54]. Administration of levetiracetam, an atypical antiepileptic agent that impedes glutamate release, reverses hippocampal-dependent memory impairments following chronic ketamine [78]. Our data are therefore consistent with existing models in which excess glutamatergic signaling promotes select learning and memory deficits [79]. Care should be taken to avoid supraphysiological prolongation of an otherwise transient increase in glutamatergic transmission following ketamine [80] or (*2R,6R*)-HNK [37], especially above and beyond that necessary to induce mechanisms underlying their persistent antidepressant effects [1, 38].

Studies have found that repeated use of ketamine (e.g., 3–4 times a week) is associated with a lasting impairment in explicit memory [13, 14, 50–52] that may be due to a sustained disruption in the function of limbic circuits that subserve memory encoding and retrieval [81]. In contrast, transient deficits in processing speed, working memory, and attention are typically observed either during or shortly after ketamine treatment at subanesthetic doses [17, 50–52, 82, 83]—which may emerge from acute disruption of higher-order executive processing [84], possibly related to prefrontal cortex connectivity [82]. Consistent with this, preclinical studies demonstrate that the hippocampus is more likely to exhibit sustained dysfunction following repeated ketamine than are higher cortical structures like the prefrontal cortex. For instance, 4 weeks of thrice-weekly ketamine (16 mg/kg, s.c.) led to a marked deficit in hippocampal-dependent delayed trace fear conditioning several months after drug cessation, whereas performance in a prefrontal cortex-dependent delayed response assay remained intact [85]. Sustained deficits in recognition memory are associated with reduced hippocampal dendritic complexity [49], also consistent with its essential role in this task [64]. Additional research is needed to better understand the broader actions of ketamine throughout corticolimbic circuits that underlie these different forms of cognition [86].

Ketamine is increasingly used as a therapeutic intervention for treatment‑resistant major depression in adolescents [87] and was recently shown to have efficacy for this indication in its first randomized active placebo-controlled clinical trial [88]. While ketamine is considered generally safe and well-tolerated in adolescent populations [89], preclinical studies suggest there may be unique consequences of early developmental exposure to ketamine when the brain is more vulnerable to prolonged psychotropic insult. This may be particularly relevant to NMDAR-inhibition-dependent actions of ketamine, since NMDAR-mediated synaptic transmission is a developmentally regulated process that is critical to the formation and maintenance of synapses in humans and rodents alike [90–92]. Indeed, early ketamine exposure can alter the trajectory of neural circuit integration through widespread changes in corticolimbic connectivity, structure, and function [53, 93–97]. While the onset of cognitive deficits tends to correspond with these circuit-level changes, our results suggest that prolonged treatment with ketamine leads to comparable recognition of memory impairment in adolescent- and adult-treated mice. Considering that studies have traditionally reported cognitive deficits within-subject, in either adolescent- or adult-treated rodents, additional studies will be needed to establish whether cognitive outcomes are truly unique to adolescent exposure, as well as the potential mechanisms that could account for any age-specific effects that are observed.

Additional work is needed to resolve the mechanism by which ketamine modulates cognitive function. Importantly, clinical status moderates the extent to which an individual will experience changes in cognition following ketamine exposure [19]. Single and repeated ketamine administration exerts pro-cognitive effects in preclinical models of depressive-like behavior [98–101] and in patients with treatment-resistant major depression [6, 21, 23, 40], especially those suffering from comorbid anxiety [40]. The cognition-enhancing properties of ketamine observed in patients suffering from depression may be explained by the fact that many of these patients show baseline cognitive deficits [24, 25] and may be related at least in part to its antidepressant actions [23, 28, 29]. In this study, however, we failed to observe pro-cognitive effects of ketamine in BALB/c mice, which exhibit anxiety-like behavior [41] and modest impairment in explicit memory (as indicated by the low discrimination index scores of control mice observed in the current study). In addition, while other NMDAR antagonists reproduce the hyperlocomotor and cognitive deficits of ketamine [102, 103], they do not share its rapid or sustained antidepressant effects [1, 104] that are mimicked by (*2R,6R*)-HNK [36, 38]. Ultimately, a better understanding of the time course along which ketamine exerts its effects on synaptic plasticity and structural remodeling is needed to foster the development of improved therapeutics that can selectively harness its benefits [1, 105].

### Conclusions

Since repeated administration of ketamine is required to sustain symptom remission, clinical studies have begun to examine what dosing frequency is sufficient to prolong its antidepressant actions. Inconsistencies in the preclinical literature have so far made it difficult to establish the cognitive effects of repeated ketamine in rodents. Here, we found that repeated ketamine exposure led to a persistent impairment of explicit memory in the novel object-recognition task and that this effect depended more on dosing frequency than on subjects’ sex or age of exposure. In contrast to our predictions, thrice-weekly ketamine led to more impairment than did daily exposure, whereas daily (*2R,6R*)-HNK led to more impairment than did thrice-weekly exposure. We also found that, while ketamine did not influence implicit memory in the passive-avoidance task, thrice-weekly (*2R,6R*)-HNK resulted in a higher avoidance of aversive stimuli whereas daily exposure led to less. This finding may be explained by an (*2R,6R*)-HNK-induced occlusion of the synaptic plasticity triggered by passive avoidance at Schaffer collateral synapses [37, 65], but more work will be needed to directly test this hypothesis. Extending these experiments to other implicit memory tasks, and using additional doses, mouse strains, and animal species, is needed to support our conclusions and may reveal additional mechanistic insights that are relevant to the unique actions of (*2R,6R*)-HNK relative to ketamine. This is especially important given that ketamine may be an effective treatment of post-traumatic stress disorder [106], a condition in which patients fail to recover from a traumatic event due to the persistence of the traumatic memory. While preclinical findings with ketamine have so far been inconsistent with regard to the extinction of learned fear [107], understanding the effects of (*2R,6R*)-HNK on threat-aggravated memory will be an important step toward understanding its potential effectiveness in stress-related disorders and its unique therapeutic mechanism of action.

## References

[1] Riggs LM, Gould TD (2021). Ketamine and the future of rapid-acting antidepressants. Annu Rev Clin Psycho.

[2] Berman RM, Cappiello A, Anand A, Oren DA, Heninger GR, Charney DS (2000). Antidepressant effects of ketamine in depressed patients. Biol Psychiat.

[3] Zarate CA, Singh JB, Carlson PJ, Brutsche NE, Ameli R, Luckenbaugh DA (2006). A randomized trial of an N-methyl-D-aspartate antagonist in treatment-resistant major depression. Arch Gen Psychiat.

[4] Murrough JW, Perez AM, Pillemer S, Stern J, Parides MK, aan het Rot M (2013). Rapid and longer-term antidepressant effects of repeated ketamine infusions in treatment-resistant major depression. Biol Psychiat.

[5] Shiroma PR, Johns B, Kuskowski M, Wels J, Thuras P, Albott CS (2014). Augmentation of response and remission to serial intravenous subanesthetic ketamine in treatment resistant depression. J Affect Disord.

[6] Shiroma PR, Thuras P, Wels J, Albott CS, Erbes C, Tye S (2020). Neurocognitive performance of repeated versus single intravenous subanesthetic ketamine in treatment resistant depression. J Affect Disord.

[7] Singh JB, Fedgchin M, Daly EJ, De Boer P, Cooper K, Lim P (2016). A double-blind, randomized, placebo-controlled, dose-frequency study of intravenous ketamine in patients with treatment-resistant depression. Am J Psychiatry.

[8] Zanos P, Moaddel R, Morris PJ, Riggs LM, Highland JN, Georgiou P (2018). Ketamine and ketamine metabolite pharmacology: insights into therapeutic mechanisms. Pharm Rev.

[9] Short B, Fong J, Galvez V, Shelker W, Loo CK (2018). Side-effects associated with ketamine use in depression: a systematic review. Lancet Psychiatry.

[10] Acevedo-Diaz EE, Cavanaugh GW, Greenstein D, Kraus C, Kadriu B, Zarate CA (2019). Comprehensive assessment of side effects associated with a single dose of ketamine in treatment-resistant depression. J Affect Disord.

[11] Phillips JL, Norris S, Talbot J, Birmingham M, Hatchard T, Ortiz A (2019). Single, repeated, and maintenance ketamine infusions for treatment-resistant depression: a randomized controlled trial. Am J Psychiat.

[12] Sassano‐Higgins S, Baron D, Juarez G, Esmaili N, Gold M (2016). A review of ketamine abuse and diversion. Depress Anxiety.

[13] Morgan CJA, Muetzelfeldt L, Curran HV (2010). Consequences of chronic ketamine self‐administration upon neurocognitive function and psychological wellbeing: a 1‐year longitudinal study. Addiction.

[14] Morgan CJA, Muetzelfeldt L, Curran HV (2009). Ketamine use, cognition and psychological wellbeing: a comparison of frequent, infrequent and ex‐users with polydrug and non‐using controls. Addiction.

[15] Zhu W, Ding Z, Zhang Y, Shi J, Hashimoto K, Lu L (2016). Risks associated with misuse of ketamine as a rapid-acting antidepressant. Neurosci Bull.

[16] Krystal JH, D'Souza DC, Karper LP, Bennett A, Abi-Dargham A, Abi-Saab D (1999). Interactive effects of subanesthetic ketamine and haloperidol in healthy humans. Psychopharmacology.

[17] Pfenninger EG, Durieux ME, Himmelseher S (2002). Cognitive impairment after small-dose ketamine isomers in comparison to equianalgesic racemic ketamine in human volunteers. Anesthesiology.

[18] Krystal JH, Karper LP, Seibyl JP, Freeman GK, Delaney R, Bremner JD (1994). Subanesthetic effects of the noncompetitive NMDA antagonist, ketamine, in humans. Arch Gen Psychiat.

[19] Souza-Marques B, Santos-Lima C, Araújo-de-Freitas L, Vieira F, Jesus-Nunes AP, Quarantini LC, et al. Neurocognitive effects of ketamine and esketamine for treatment-resistant major depressive disorder: a systematic review. Harvard Rev Psychiat. 2021; 10.1097/hrp.0000000000000312. 10.1097/HRP.0000000000000312 34366408

[20] Araújo-De-Freitas L, Santos-Lima C, Mendonça-Filho E, Vieira F, França RJAF, Magnavita G (2021). Neurocognitive aspects of ketamine and esketamine on subjects with treatment-resistant depression: a comparative, randomized and double-blind study. Psychiat Res.

[21] Gill H, Gill B, Rodrigues NB, Lipsitz O, Rosenblat JD, El-Halabi S (2020). The effects of ketamine on cognition in treatment-resistant depression: a systematic review and priority avenues for future research. Neurosci Biobehav Rev.

[22] Crisanti C, Enrico P, Fiorentini A, Delvecchio G, Brambilla P (2020). Neurocognitive impact of Ketamine treatment in major depressive disorder: a review on human and animal studies. J Affect Disord.

[23] Shiroma PR, Albott CS, Johns B, Thuras P, Wels J, Lim KO (2014). Neurocognitive performance and serial intravenous subanesthetic ketamine in treatment-resistant depression. Int J Neuropsychoph.

[24] Conradi HJ, Ormel J, de Jonge P (2011). Presence of individual (residual) symptoms during depressive episodes and periods of remission: a 3-year prospective study. Psychol Med.

[25] Perini G, Ramusino MC, Sinforiani E, Bernini S, Petrachi R, Costa A (2019). Cognitive impairment in depression: recent advances and novel treatments. Neuropsych Dis Treat.

[26] Lee RSC, Hermens DF, Porter MA, Redoblado-Hodge MA (2012). A meta-analysis of cognitive deficits in first-episode major depressive disorder. J Affect Disord.

[27] Bora E, Harrison BJ, Yücel M, Pantelis C (2013). Cognitive impairment in euthymic major depressive disorder: a meta-analysis. Psychol Med.

[28] Murrough JW, Wan LB, Iacoviello B, Collins KA, Solon C, Glicksberg B (2014). Neurocognitive effects of ketamine in treatment-resistant major depression: association with antidepressant response. Psychopharmacology.

[29] Murrough JW, Burdick KE, Levitch CF, Perez AM, Brallier JW, Chang LC (2015). Neurocognitive effects of ketamine and association with antidepressant response in individuals with treatment-resistant depression: a randomized controlled trial. Neuropsychopharmacology.

[30] Diamond PR, Farmery AD, Atkinson S, Haldar J, Williams N, Cowen PJ (2014). Ketamine infusions for treatment resistant depression: a series of 28 patients treated weekly or twice weekly in an ECT clinic. J Psychopharmacol.

[31] Kessling LV (1998). Cognitive impairment in the euthymic phase of affective disorder. Psychol Med.

[32] Strong CE, Kabbaj M (2018). On the safety of repeated ketamine infusions for the treatment of depression: effects of sex and developmental periods. Neurobiol Stress.

[33] Neill JC, Barnes S, Cook S, Grayson B, Idris NF, McLean SL (2010). Animal models of cognitive dysfunction and negative symptoms of schizophrenia: Focus on NMDA receptor antagonism. Pharm Therapeut.

[34] Young JW, Powell SB, Risbrough V, Marston HM, Geyer MA (2009). Using the MATRICS to guide development of a preclinical cognitive test battery for research in schizophrenia. Pharm Therapeut.

[35] Lumsden EW, Troppoli TA, Myers SJ, Zanos P, Aracava Y, Kehr J (2019). Antidepressant-relevant concentrations of the ketamine metabolite (*2R,6R*)-hydroxynorketamine do not block NMDA receptor function. Proc Natl Acad Sci USA.

[36] Zanos P, Moaddel R, Morris PJ, Georgiou P, Fischell J, Elmer GI (2016). NMDAR inhibition-independent antidepressant actions of ketamine metabolites. Nature.

[37] Riggs LM, Aracava Y, Zanos P, Fischell J, Albuquerque EX, Pereira EFR, et al. (*2R,6R*)-hydroxynorketamine rapidly potentiates hippocampal glutamatergic transmission through a synapse-specific presynaptic mechanism. Neuropsychopharmacology. 2020;45:426–36. 10.1038/s41386-019-0443-3 PMC6901515 31216563

[38] Highland JN, Zanos P, Riggs LM, Georgiou P, Clark SM, Morris PJ (2021). Hydroxynorketamines: pharmacology and potential therapeutic applications. Pharm Rev.

[39] Morris PJ, Moaddel R, Zanos P, Moore CE, Gould TD, Zarate CA (2017). Synthesis and N-methyl-D-aspartate (NMDA) receptor activity of ketamine metabolites. Org Lett.

[40] Liu W, Zhou Y, Zheng W, Wang C, Zhan Y, Lan X (2019). Repeated intravenous infusions of ketamine: neurocognition in patients with anxious and nonanxious treatment-resistant depression. J Affect Disord.

[41] Sartori SB, Landgraf R, Singewald N (2011). The clinical implications of mouse models of enhanced anxiety. Futur Neurol.

[42] Webster SJ, Bachstetter AD, Nelson PT, Schmitt FA, Eldik LJV (2014). Using mice to model Alzheimer’s dementia: an overview of the clinical disease and the preclinical behavioral changes in 10 mouse models. Front Genet.

[43] Beltz AM, Beery AK, Becker JB (2019). Analysis of sex differences in pre-clinical and clinical data sets. Neuropsychopharmacology.

[44] Kryst J, Kawalec P, Mitoraj AM, Pilc A, Lasoń W, Brzostek T (2020). Efficacy of single and repeated administration of ketamine in unipolar and bipolar depression: a meta-analysis of randomized clinical trials. Pharm Rep..

[45] Morgan CJA, Curran HV (2006). Acute and chronic effects of ketamine upon human memory: a review. Psychopharmacology.

[46] Hauser MJ, Isbrandt D, Roeper J (2017). Disturbances of novel object exploration and recognition in a chronic ketamine mouse model of schizophrenia. Behav Brain Res.

[47] Neves G, Borsoi M, Antonio CB, Pranke MA, Betti AH, Rates S (2017). Is forced swimming immobility a good endpoint for modeling negative symptoms of schizophrenia? Study of sub-anesthetic ketamine repeated administration effects. An Da Acad Brasileira De Ciências.

[48] Jacklin DL, Goel A, Clementino KJ, Hall AW, Talpos JC, Winters BD (2012). Severe cross-modal object recognition deficits in rats treated sub-chronically with NMDA receptor antagonists are reversed by systemic nicotine: implications for abnormal multisensory integration in schizophrenia. Neuropsychopharmacology.

[49] Suárez-Santiago JE, Orozco-Suárez S, Vega-García A, Bautista-Orozco LÁ, Picazo O (2020). Repeated ketamine administration induces recognition memory impairment together with morphological changes in neurons from ventromedial prefrontal cortex, dorsal striatum, and hippocampus. Behav Pharm.

[50] Newcomer JW, Farber NB, Jevtovic-Todorovic V, Selke G, Melson AK, Hershey T (1999). Ketamine-induced NMDA receptor hypofunction as a model of memory impairment and psychosis. Neuropsychopharmacology.

[51] Morgan CJA, Mofeez A, Brandner B, Bromley L, Curran HV (2004). Acute effects of ketamine on memory systems and psychotic symptoms in healthy volunteers. Neuropsychopharmacology.

[52] Malhotra AK, Pinals DA, Weingartner H, Sirocco K, Missar CD, Pickar D (1996). NMDA receptor function and human cognition: the effects of ketamine in healthy volunteers. Neuropsychopharmacology.

[53] Nagy LR, Featherstone RE, Hahn CG, Siegel SJ (2015). Delayed emergence of behavioral and electrophysiological effects following juvenile ketamine exposure in mice. Transl Psychiat.

[54] Featherstone RE, Liang Y, Saunders JA, Tatard-Leitman VM, Ehrlichman RS, Siegel SJ (2012). Subchronic ketamine treatment leads to permanent changes in EEG, cognition and the astrocytic glutamate transporter EAAT2 in mice. Neurobiol Dis.

[55] Célia Moreira Borella V, Seeman MV, Carneiro Cordeiro R, Vieira dos Santos J, Romário Matos de Souza M, Nunes de Sousa Fernandes E (2016). Gender and estrous cycle influences on behavioral and neurochemical alterations in adult rats neonatally administered ketamine. Dev Neurobiol.

[56] Nikiforuk A, Fijał K, Potasiewicz A, Popik P, Kos T (2013). The 5-hydroxytryptamine (serotonin) receptor 6 agonist EMD 386088 ameliorates ketamine-induced deficits in attentional set shifting and novel object recognition, but not in the prepulse inhibition in rats. J Psychopharmacol.

[57] Nikiforuk A, Popik P (2012). Effects of quetiapine and sertindole on subchronic ketamine-induced deficits in attentional set-shifting in rats. Psychopharmacology.

[58] Nikiforuk A, Popik P (2014). The effects of acute and repeated administration of ketamine on attentional performance in the five-choice serial reaction time task in rats. Eur Neuropsychopharm.

[59] Wang JH, Fu Y, Wilson FAW, Ma YY (2006). Ketamine affects memory consolidation: differential effects in T-maze and passive avoidance paradigms in mice. Neuroscience.

[60] Popik P, Hołuj M, Kos T, Nowak G, Librowski T, Sałat K (2017). Comparison of the psychopharmacological effects of tiletamine and ketamine in rodents. Neurotox Res.

[61] Zugno AI, Matos MP, Canever L, Fraga DB, De Luca RD, Ghedim FV (2014). Evaluation of acetylcholinesterase activity and behavioural alterations induced by ketamine in an animal model of schizophrenia. Acta Neuropsychiatr.

[62] Winters BD, Forwood SE, Cowell RA, Saksida LM, Bussey TJ (2004). Double dissociation between the effects of peri-postrhinal cortex and hippocampal lesions on tests of object recognition and spatial memory: heterogeneity of function within the temporal lobe. J Neurosci.

[63] Cohen SJ, Munchow AH, Rios LM, Zhang G, Asgeirsdóttir HN, Stackman RW (2013). The rodent hippocampus is essential for nonspatial object memory. Curr Biol Cb.

[64] Cohen SJ, Stackman RW (2015). Assessing rodent hippocampal involvement in the novel object recognition task. A review. Behav Brain Res.

[65] Whitlock JR, Heynen AJ, Shuler MG, Bear MF (2006). Learning induces long-term potentiation in the hippocampus. Science.

[66] Chou D (2020). Topiramate inhibits offensive aggression through targeting ventrolateral periaqueductal gray. Neuropharmacology.

[67] Chou D (2020). Brain-derived neurotrophic factor in the ventrolateral periaqueductal gray contributes to (*2R,6R*)-hydroxynorketamine-mediated actions. Neuropharmacology.

[68] Ye L, Ko CY, Huang Y, Zheng C, Zheng Y, Chou D (2019). Ketamine metabolite (*2R,6R*)-hydroxynorketamine enhances aggression via periaqueductal gray glutamatergic transmission. Neuropharmacology.

[69] Luo, Y, Yu, Y, Zhang, M, et al. Chronic administration of ketamine induces cognitive deterioration by restraining synaptic signaling. Mol Psychiatry (2020). 10.1038/s41380-020-0793-6. 10.1038/s41380-020-0793-6 32488127

[70] Hsieh C-P, Chen S-T, Lee M-Y, Huang C-M, Chen H-H, Chan M-H, et al. N-dimethylglycine protects behavioral disturbances and synaptic deficits induced by repeated ketamine exposure in mice. Neuroscience. 2021;460:88–106. 10.1016/j.neuroscience.2021.08.004 34400248

[71] Shaffer CL, Osgood SM, Smith DL, Liu J, Trapa PE (2014). Enhancing ketamine translational pharmacology via receptor occupancy normalization. Neuropharmacology.

[72] Rajagopal L, Massey B, Huang M, Oyamada Y, Meltzer H (2014). The novel object recognition test in rodents in relation to cognitive impairment in schizophrenia. Curr Pharm Des.

[73] Crawford CA, Moran AE, Baum TJ, Apodaca MG, Montejano NR, Park GI (2020). Effects of monoamine depletion on the ketamine-induced locomotor activity of preweanling, adolescent, and adult rats: sex and age differences. Behav Brain Res.

[74] Bates MLS, Trujillo KA (2019). Long-lasting effects of repeated ketamine administration in adult and adolescent rats. Behav Brain Res.

[75] Trujillo KA, Heller CY (2020). Ketamine sensitization: Influence of dose, environment, social isolation and treatment interval. Behav Brain Res.

[76] McDougall SA, Moran AE, Baum TJ, Apodaca MG, Real V (2017). Effects of ketamine on the unconditioned and conditioned locomotor activity of preadolescent and adolescent rats: impact of age, sex, and drug dose. Psychopharmacology.

[77] Schoepfer KJ, Strong CE, Saland SK, Wright KN, Kabbaj M (2019). Sex- and dose-dependent abuse liability of repeated subanesthetic ketamine in rats. Physiol Behav.

[78] Koh MT, Shao Y, Rosenzweig-Lipson S, Gallagher M (2018). Treatment with levetiracetam improves cognition in a ketamine rat model of schizophrenia. Schizophr Res.

[79] Wenk GL, Parsons CG, Danysz W (2006). Potential role of N-methyl-D-aspartate receptors as executors of neurodegeneration resulting from diverse insults: focus on memantine. Behav Pharm.

[80] Moghaddam B, Adams B, Verma A, Daly D (1997). Activation of glutamatergic neurotransmission by ketamine: a novel step in the pathway from NMDA receptor blockade to dopaminergic and cognitive disruptions associated with the prefrontal cortex. J Neurosci.

[81] Honey GD, Honey RA, O'Loughlin C, Sharar SR, Kumaran D, Suckling J (2005). Ketamine disrupts frontal and hippocampal contribution to encoding and retrieval of episodic memory: an fMRI study. Cereb Cortex.

[82] Driesen NR, McCarthy G, Bhagwagar Z, Bloch MH, Calhoun VD, D'Souza DC (2013). The impact of NMDA receptor blockade on human working memory-related prefrontal function and connectivity. Neuropsychopharmacology.

[83] Ghoneim MM, Hinrichs JV, Mewaldt SP, Petersen RC (1985). Ketamine. J Clin Psychopharm.

[84] Honey RA, Turner DC, Honey GD, Sharar SR, Kumaran D, Pomarol-Clotet E (2003). Subdissociative dose ketamine produces a deficit in manipulation but not maintenance of the contents of working memory. Neuropsychopharmacology.

[85] Koh MT, Shao Y, Sherwood A, Smith DR (2016). Impaired hippocampal-dependent memory and reduced parvalbumin-positive interneurons in a ketamine mouse model of schizophrenia. Schizophr Res.

[86] Price RB, Duman R (2020). Neuroplasticity in cognitive and psychological mechanisms of depression: an integrative model. Mol Psychiatr.

[87] Kim S, Rush BS, Rice TR. A systematic review of therapeutic ketamine use in children and adolescents with treatment-resistant mood disorders. Eur Child Adoles Psy. 2021;1485-1501. 10.1007/s00787-020-01542-3. 10.1007/s00787-020-01542-3 32385697

[88] Dwyer JB, Landeros-Weisenberger A, Johnson JA, Londono Tobon A, Flores JM, Nasir M (2021). Efficacy of intravenous ketamine in adolescent treatment-resistant depression: a randomized midazolam-controlled trial. Am J Psychiat.

[89] Di Vincenzo JD, Siegel A, Lipsitz O, Ho R, Teopiz KM, Ng J (2021). The effectiveness, safety and tolerability of ketamine for depression in adolescents and older adults: a systematic review. J Psychiatr Res.

[90] Law AJ, Weickert CS, Webster MJ, Herman MM, Kleinman JE, Harrison PJ (2003). Expression of NMDA receptor NR1, NR2A and NR2B subunit mRNAs during development of the human hippocampal formation. Eur J Neurosci.

[91] Wenzel A, Fritschy JM, Mohler H, Benke D (1997). NMDA receptor heterogeneity during postnatal development of the rat brain: differential expression of the NR2A, NR2B, and NR2C subunit proteins. J Neurochem.

[92] Sans N, Petralia RS, Wang YX, Blahos J, Hell JW, Wenthold RJ (2000). A developmental change in NMDA receptor-associated proteins at hippocampal synapses. J Neurosci.

[93] Featherstone RE, Nagy LR, Hahn CG, Siegel SJ (2014). Juvenile exposure to ketamine causes delayed emergence of EEG abnormalities during adulthood in mice. Drug Alcohol Depen.

[94] Jeevakumar V, Driskill C, Paine A, Sobhanian M, Vakil H, Morris B (2015). Ketamine administration during the second postnatal week induces enduring schizophrenia-like behavioral symptoms and reduces parvalbumin expression in the medial prefrontal cortex of adult mice. Behav Brain Res.

[95] Zhang H, Sun XR, Wang J, Zhang ZZ, Zhao HT, Li HH (2016). Reactive oxygen species-mediated loss of phenotype of parvalbumin interneurons contributes to long-term cognitive impairments after repeated neonatal ketamine exposures. Neurotox Res.

[96] Pérez MÁ, Morales C, Santander O, García F, Gómez I, Peñaloza-Sancho V (2019). Ketamine-treatment during late adolescence impairs inhibitory synaptic transmission in the prefrontal cortex and working memory in adult rats. Front Cell Neurosci.

[97] Onaolapo AY, Ayeni OJ, Ogundeji MO, Ajao A, Onaolapo OJ, Owolabi AR (2019). Subchronic ketamine alters behaviour, metabolic indices and brain morphology in adolescent rats: Involvement of oxidative stress, glutamate toxicity and caspase-3-mediated apoptosis. J Chem Neuroanat.

[98] Aleksandrova LR, Wang YT, Phillips AG (2020). Ketamine and its metabolite, (2R,6R)-HNK, restore hippocampal LTP and long-term spatial memory in the Wistar-Kyoto rat model of depression. Mol Brain.

[99] Willner P, Gruca P, Lason M, Tota-Glowczyk K, Litwa E, Niemczyk M (2018). Validation of chronic mild stress in the Wistar-Kyoto rat as an animal model of treatment-resistant depression. Behav Pharm.

[100] Papp M, Gruca P, Lason-Tyburkiewicz M, Willner P (2017). Antidepressant, anxiolytic and procognitive effects of subacute and chronic ketamine in the chronic mild stress model of depression. Behav Pharm.

[101] Yang Y, Ju W, Zhang H, Sun L (2018). Effect of ketamine on LTP and NMDAR EPSC in hippocampus of the chronic social defeat stress mice model of depression. Front Behav Neurosci.

[102] Haaf M, Leicht G, Curic S, Mulert C (2018). Glutamatergic deficits in schizophrenia—biomarkers and pharmacological interventions within the ketamine model. Curr Pharm Biotechnol.

[103] Cadinu D, Grayson B, Podda G, Harte MK, Doostdar N, Neill JC (2018). NMDA receptor antagonist rodent models for cognition in schizophrenia and identification of novel drug treatments, an update. Neuropharmacology.

[104] Gould TD, Zarate JCA, Thompson SM (2017). Molecular pharmacology and neurobiology of rapid-acting antidepressants. Annu Rev Pharm.

[105] Wu H, Savalia NK, Kwan AC (2021). Ketamine for a boost of neural plasticity: how, but also when?. Biol Psychiat.

[106] Feder A, Rutter SB, Schiller D, Charney DS (2020). The emergence of ketamine as a novel treatment for posttraumatic stress disorder. Adv Pharm.

[107] Choi KH, Berman RY, Zhang M, Spencer HF, Radford KD (2020). Effects of ketamine on rodent fear memory. Int J Mol Sci.

